# Fixation height has a greater biomechanical effect than anterior tilt angle in syndesmotic fixation for weber B ankle fractures: a specific finite element study

**DOI:** 10.3389/fsurg.2026.1890743

**Published:** 2026-07-14

**Authors:** Dong Jiang, Zhiwei Wu, Jianwen Cheng

**Affiliations:** 1 Department of Orthopaedic Traumatology and Hand Surgery, The First Affiliated Hospital to Guangxi Medical University, Nanning, Guangxi, China; 2 Department of Orthopedics, Loudi Central Hospital, Loudi, Hunan, China; 3 Department of 120 Emergency Station, Loudi Central Hospital, Loudi, Hunan, China

**Keywords:** ankle fracture, biomechanics, finite element analysis, suture-button, syndesmosis, tricortical screw

## Abstract

**Background:**

Distal tibiofibular syndesmotic instability in Weber B ankle fractures remains challenging, as fixation constructs and insertion parameters alter ankle biomechanics.

**Methods:**

A 3D finite element ankle model was developed from CT/MRI data of a healthy adult. Residual instability was simulated by transecting the anterior inferior tibiofibular and interosseous ligaments after anatomical reduction and fixation of all malleolar fractures. Eighteen fixation models were created using tricortical screws or suture-buttons, placed 20, 30, or 40 mm above the tibial plafond with anterior tilt angles of 12.5°, 27.5°, or 42.5°. Outcomes included peak tibiotalar contact stress, fibular displacement, and peak implant von Mises stress under axial loading and external rotation.

**Results:**

Residual instability increased peak tibiotalar contact stress by 36.8% and markedly increased fibular translation. Fixation at 20–30 mm restored near-physiologic mechanics, while 40 mm fixation showed biomechanical deterioration. At equivalent heights, tricortical screws better restrained fibular translation, whereas suture-buttons preserved more micromotion but had higher implant stress concentrations.

**Conclusions:**

Fixation height had a larger biomechanical effect than anterior tilt angle. Fixation 20–30 mm above the tibial plafond is recommended, with construct selection balancing rigidity and physiologic syndesmotic motion.

## 1 Introduction

Ankle fractures account for a substantial proportion of musculoskeletal injuries, and Weber B fractures are among the most common patterns encountered in clinical practice (1, 2). In these injuries, the distal tibiofibular syndesmosis is a major determinant of ankle congruity, load transfer, and long-term joint preservation (3, 4). The syndesmotic complex, composed primarily of the anterior inferior tibiofibular ligament (AITFL), posterior inferior tibiofibular ligament (PITFL), interosseous ligament (IOL), and transverse tibiofibular ligament（TTFL）, constrains the fibula while still permitting small physiologic translations and rotations during ankle motion (5–7). When this stabilizing complex is injured and not restored accurately, even subtle talar or fibular malposition can substantially increase contact stress and accelerate post-traumatic osteoarthritis (4, 8, 9).

The standard treatment for unstable syndesmotic injuries is surgical fixation, but important technical questions remain unresolved. Tricortical or quadricortical syndesmotic screw fixation has long been considered the traditional reference technique because it provides strong initial stability and is technically straightforward (9, 10). However, screw fixation may overconstrain physiologic syndesmotic motion and is associated with screw loosening, breakage, and possible need for implant removal (11, 12). Suture-button fixation was introduced to provide dynamic stabilization while preserving limited physiologic micromotion (13). Clinical studies and meta-analyses have suggested that suture-button fixation may improve early recovery and reduce reoperation rates in selected patients (11–13), although concerns remain regarding rotational stability and implant durability, especially under high-demand loading conditions (14).

Beyond implant choice, the technical details of syndesmotic fixation may also influence outcome. Fixation height relative to the tibial plafond, drill anterior tilt, and cortical purchase have all been discussed in cadaveric and clinical studies, but consensus remains incomplete (15, 16). AO-based recommendations generally favor placement approximately 20–30 mm above the ankle joint line, yet quantitative evidence comparing 20, 30, and 40 mm fixation planes remains limited (17). Likewise, the biomechanical consequences of different sagittal insertion trajectories have not been systematically defined, particularly for suture-button constructs.

Finite element analysis (FEA) provides an opportunity to evaluate these questions in a controlled, patient-specific environment. Prior ankle FEA studies have shown that subject-specific models can reliably predict joint contact mechanics and chronic stress exposure (18, 19). However, few studies have comprehensively compared syndesmotic fixation construct, fixation height, and anterior tilt angle in a Weber B injury setting. Therefore, the purpose of this study was to compare tricortical screw and suture-button fixation across three fixation heights and three anterior tilt angles in a patient-specific finite element model of Weber B ankle fracture with residual syndesmotic instability. We hypothesized that: (1) residual syndesmotic instability would increase tibiotalar contact stress and fibular displacement; (2) both fixation methods would improve biomechanics, with screws providing greater restraint and suture-buttons preserving more micromotion; and (3) under the tested loading and injury conditions, fixation height would influence ankle biomechanics more strongly than anterior tilt angle, with 20–30 mm placement outperforming 40 mm placement.

## 2 Materials and methods

### 2.1 Imaging acquisition and model reconstruction

A single-subject patient-specific ankle model was created from CT and MRI data obtained from the right ankle of one healthy 27-year-old male volunteer. It should be emphasized that only the osseous and cartilaginous geometry of this model was subject-specific, while all material properties, ligament mechanical behaviors, and loading conditions were derived from published literature and standardized assumptions, not measured from this individual. This imaging-based study was approved by the Institutional Review Board of The First Affiliated Hospital of Guangxi Medical University (2025-K0456), and written informed consent was obtained from the volunteer before imaging.

**Figure 1:**
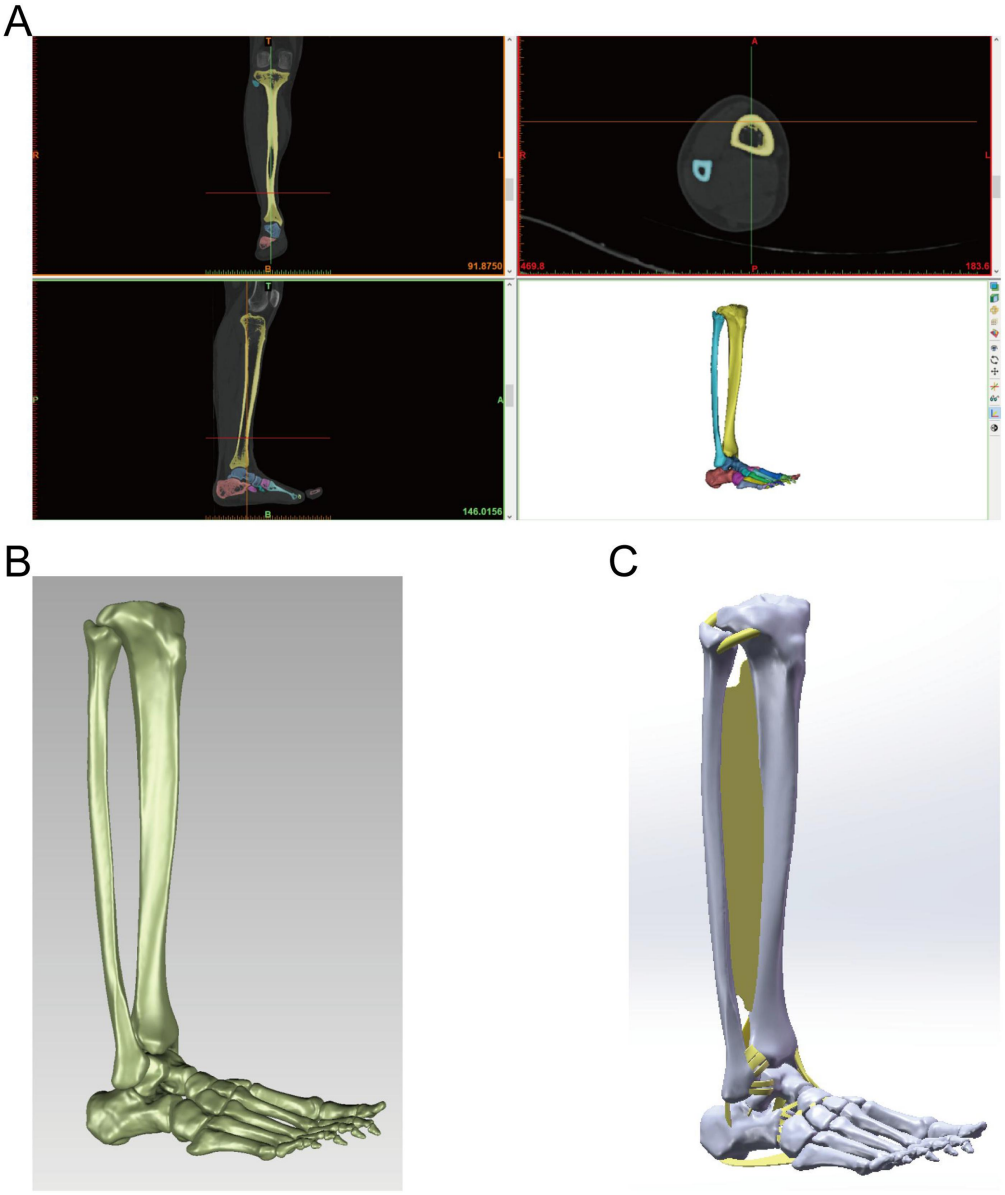
Workflow of finite element model generation. **(A)** Geometric reconstruction and primary topology processing. **(B)** Surface solid model. **(C)** Complete Three-Dimensional Solid Models.

High-resolution CT data were used to reconstruct the osseous structures, including the tibia, fibula, talus, and calcaneus. MRI data from the same ankle were used to define articular cartilage geometry and ligament attachment sites. DICOM data were segmented in Mimics 19.0 (Materialise, Leuven, Belgium), and the resulting surface models were optimized in Geomagic Wrap 2017 (3D Systems, Rock Hill, SC, USA). Solid models were created and assembled in SolidWorks (Dassault Systèmes, Waltham, MA, USA), and finite element simulations were performed in ANSYS Mechanical (ANSYS Inc., Canonsburg, PA, USA). The final model included the tibia, fibula, talus, calcaneus, distal tibial and talar cartilage, and the major ligamentous stabilizers relevant to ankle and syndesmotic mechanics. These included the AITFL, PITFL, IOL, TTFL, ATFL, PTFL, CFL, deltoid ligament complex, interosseous membrane, and proximal tibiofibular ligaments(Figure 1 A–C).

### 2.2 Injury model and fixation constructs

To isolate the independent biomechanical role of syndesmotic fixation and eliminate confounding effects from malleolar fracture instability, the all malleolus fractures were assumed to have been anatomically reduced and rigidly stabilized with a lateral fibular plate and hollow screws, leaving residual instability at the syndesmosis as the principal variable of interest. This simplification was adopted to focus specifically on syndesmotic fixation mechanics, which is a common approach in comparative biomechanical studies of syndesmotic injuries. A Weber B-equivalent injury condition was simulated by transection of the AITFL and IOL. It is acknowledged that clinical Weber B fractures may involve additional injuries to the posterior inferior tibiofibular ligament (PITFL), deltoid ligament, or variable osseous instability, which were not included in the present model.

A total of 20 models were analyzed:

1. intact model;
2. injured model with residual syndesmotic instability;
3. tricortical screw fixation models;
4. suture-button fixation models.

**Figure 2:**
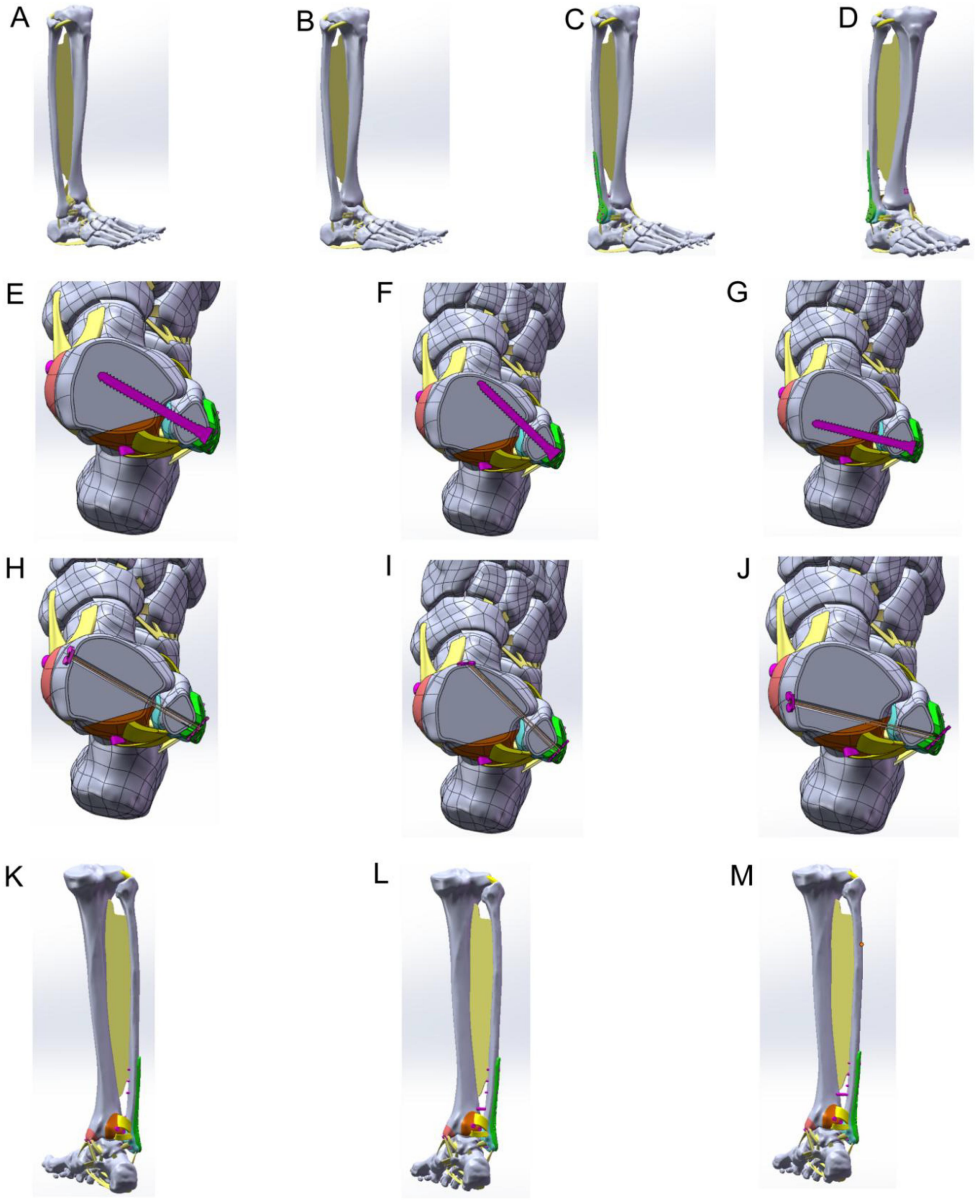
Three-Dimensional solid models: **(A)** control group; **(B)** tibiofibular syndesmotic injury; **(C)** simulated fracture internal fixation and single tricortical screw; **(D)** simulated fracture internal fixation and suture-button; **(E–G)** three anteversion insertion methods of tri-cortical screw; **(H–J)** three anteversion angle implantation methods for S-B fixation; **(K–M)** peg placement model with different heights.

For both fixation methods, implants were placed at 20, 30, or 40 mm proximal to the tibial plafond with anterior tilt angles of 12.5°, 27.5°, or 42.5°. The three sagittal anterior tilt angles (12.5°, 27.5°, 42.5°) were selected to cover the typical range of insertion trajectories encountered in clinical practice, based on retrospective CT measurements of syndesmotic screw placement reported in prior studies (15, 20). The midpoint value of 27.5° approximates the average anterior tilt angle used in standard surgical techniques, while 12.5° and 42.5° represent the lower and upper bounds of clinically acceptable insertion angles to avoid intra-articular penetration or posterior cortical violation. It should be noted that this range may not capture all possible extreme trajectories encountered in clinical practice. All fixation models displayed in Table 1 and assumed that all malleolus fractures had been anatomically reduced and stabilized with a lateral fibular plate and hollow screws; only the syndesmotic fixation strategy, fixation height, and anterior tilt angle were varied**(**Figure 2 A–M**)**.

**Table 1:** Finite element model groups and parametric design of syndesmotic fixation.

Model code	Construct	Fixation height above tibial plafond (mm)	Sagittal anterior tilt angle (°)	Model description
Intact	None	–	–	Intact ankle model
Injured	None	–	–	Residual syndesmotic instability model with AITFL and IOL transection
A1	Single tricortical screw	20	27.5	Screw fixation model
A2	Single tricortical screw	20	42.5	Screw fixation model
A3	Single tricortical screw	20	12.5	Screw fixation model
C1	Single tricortical screw	30	27.5	Screw fixation model
C2	Single tricortical screw	30	42.5	Screw fixation model
C3	Single tricortical screw	30	12.5	Screw fixation model
E1	Single tricortical screw	40	27.5	Screw fixation model
E2	Single tricortical screw	40	42.5	Screw fixation model
E3	Single tricortical screw	40	12.5	Screw fixation model
B1	Suture-button	20	27.5	Suture-button fixation model
B2	Suture-button	20	42.5	Suture-button fixation model
B3	Suture-button	20	12.5	Suture-button fixation model
D1	Suture-button	30	27.5	Suture-button fixation model
D2	Suture-button	30	42.5	Suture-button fixation model
D3	Suture-button	30	12.5	Suture-button fixation model
F1	Suture-button	40	27.5	Suture-button fixation model
F2	Suture-button	40	42.5	Suture-button fixation model
F3	Suture-button	40	12.5	Suture-button fixation model

### 2.3 Mesh generation, material assignment, and contact definitions

The assembled models were meshed predominantly with 10-node tetrahedral elements. Local mesh refinement was applied to the tibiotalar contact region, syndesmotic region, ligament insertions, and implant interfaces to improve numerical stability and capture stress gradients. A formal mesh convergence analysis was not performed in the present study, which represents a limitation. However, the mesh density and refinement strategy used were consistent with previously validated finite element models of the ankle joint (18, 19), which have been shown to produce reliable contact stress and displacement results. A comprehensive mesh convergence study will be included in future iterations of this work.

All tissues and implants were modeled as homogeneous, isotropic, linear elastic materials to facilitate convergence in the nonlinear contact simulations. This is a standard simplification in comparative finite element studies of orthopedic implants, but it has important limitations: biological tissues such as ligaments, cartilage, and bone exhibit inherent anisotropy, nonlinearity, and viscoelasticity. In particular, ligaments demonstrate nonlinear tensile behavior and cannot resist compression, while ultra-high-molecular-weight polyethylene (UHMWPE) sutures behave as tensile-only flexible materials rather than linear elastic solids. These simplifications may overestimate the stiffness of ligamentous and suture structures and alter the predicted load-sharing patterns between implants and native tissues. A sensitivity analysis incorporating nonlinear material models is planned for future studies to quantify the impact of these assumptions on the results.

Material properties for cortical bone, cancellous bone, cartilage, titanium alloy implants, and UHMWPE suture material were assigned from published literature and are summarized in Tables 2, 3.

**Table 2:** Material properties assigned to bone, cartilage, and implant components.

Component/material	Elastic modulus, E (MPa)	Poisson's ratio, *ν*	Description
Cortical bone	17,500	0.30	Macro-scale value for dense cortical bone
Cancellous bone	1,500	0.20	Lower modulus reflecting porous trabecular architecture
Articular cartilage	12.0	0.49	Near-incompressible cartilage behavior
Titanium alloy implant (Ti-6Al-4 V)	110,000	0.30	Plate, screw, and metallic button components
UHMWPE suture material	1,000	0.35	Suture-button fiber component

**Table 3:** Material properties assigned to ligament structures included in the finite element model.

Ligament structure	Elastic modulus, E (MPa)	Reference stiffness (N/m)
AITFL	78.0	13.0
PITFL	101.0	16.8
TTFL	70.0	14.0
IOL	224.2	24.9
ATFL	39.9	8.0
PTFL	39.9	8.0
CFL	70.5	11.8
Interosseous talocalcaneal ligament	70.0	14.0
Medial talocalcaneal ligament	70.0	10.0
Lateral talocalcaneal ligament	70.0	11.7
Posterior talocalcaneal ligament	70.0	14.0
Anterior talocalcaneal (cervical) ligament	70.0	14.0
Deltoid ligament	128.8	5.6
Interosseous membrane	224.2	4.3
Anterior proximal tibiofibular ligament	133.0	26.6
Posterior proximal tibiofibular ligament	109.0	15.6

Cartilage-cartilage interactions were defined as frictional contact with a low coefficient of friction to simulate lubricated joint articulation. Ligament-bone and cartilage-bone interfaces were modeled as bonded. Screw-bone, plate-bone, and implant interfaces were also modeled as bonded to reflect rigid, stable fixation in the immediate postoperative period (0–2 weeks), when biological osseointegration has not yet occurred and implant stability relies entirely on mechanical purchase. This assumption does not account for potential micromotion, loosening, or osseointegration that may occur during long-term follow-up, which could alter implant stress distribution and construct stability over time.

### 2.4 Boundary conditions and outcome measures

To simulate the biomechanical environment of early postoperative partial weight-bearing with external rotation stress (corresponding to approximately 2–4 weeks after surgery, when patients typically begin partial weight-bearing with protected mobilization), the proximal tibia was fixed in all degrees of freedom. An axial compressive load of 200 N (representing ∼25% of body weight for an 80 kg individual) was applied to the calcaneus, and an 5N.m external rotation moment was applied to the talus with the ankle held in a neutral position. This loading combination has been widely used in prior biomechanical studies of syndesmotic fixation to simulate the most critical stress state for construct failure (14, 16). The stability of the conclusions under different load intensities (e.g., full weight-bearing, higher external rotation moments) should be evaluated in future studies.

**Figure 3:**
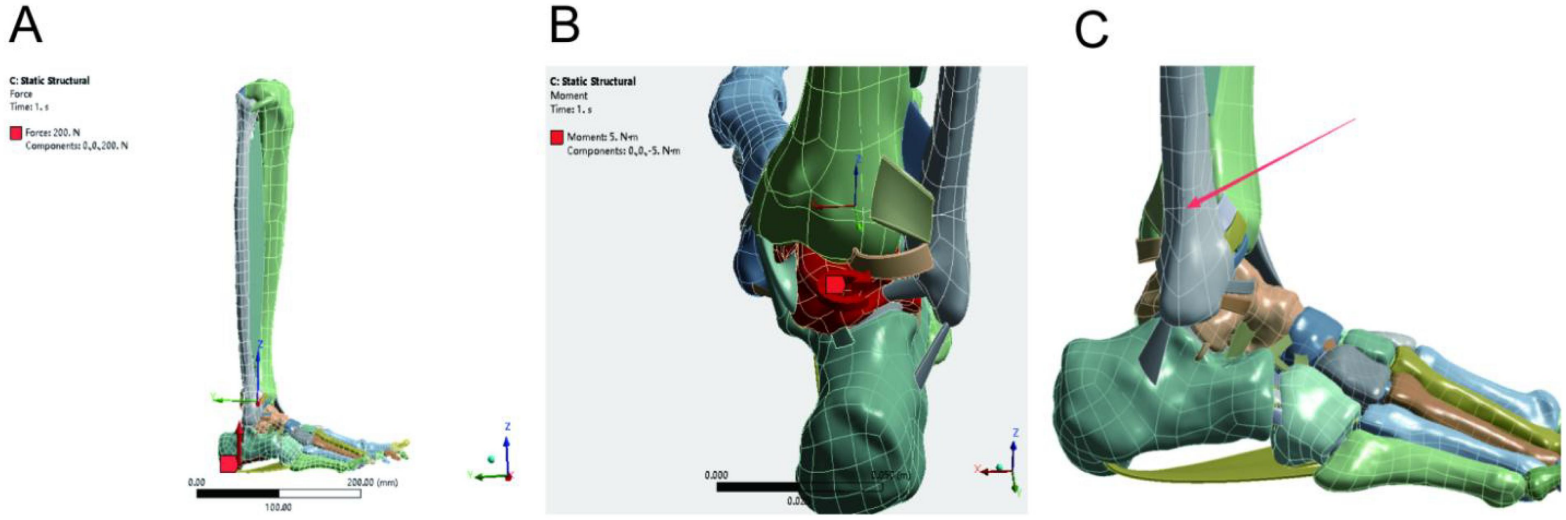
Boundary conditions, loading protocol, and coordinate system used for finite element analysis. **(A)** The proximal tibia was constrained, and an axial compressive load of 200 N was applied through the calcaneus to simulate partial weight-bearing. **(B)** An external rotation moment was applied to the talus with the ankle maintained in a neutral position. **(C)** A local coordinate system was established to quantify distal fibular motion.

A local coordinate system was defined to quantify distal fibular motion. Lateral displacement was measured along the *x*-axis and posterior displacement along the *y*-axis at the most prominent point on the lateral cortex located 10 mm proximal to the distal fibular articular surface (Figure 3 A–C).

For the purposes of this study, “physiologic micromotion” was defined as the range of distal fibular displacement observed in the intact ankle model under the same loading conditions (1.20 mm lateral displacement and 3.69 mm posterior displacement). A “dominant biomechanical effect” was defined as a change in an outcome measure of at least 50% when varying one parameter, compared to less than 20% change when varying the other parameter within their respective tested ranges.

### 2.5 Model validation

Before pathologic conditions and implants were introduced, the intact model was validated under 600 N axial compression, which is a standard validation protocol for ankle finite element models. Predicted peak tibiotalar contact stress was compared with previously published cadaveric and finite element data reported by Anderson et al. (21), which is the most widely cited benchmark for ankle contact mechanics. It is acknowledged that validation was limited to peak tibiotalar contact stress in the intact model, and additional validation of distal fibular displacement and implant stress against experimental data was not performed. This represents a limitation of the present study, and future work will include validation of these key outcome measures using cadaveric testing data.

## 3 Results

### 3.1 Model validation

**Figure 4:**
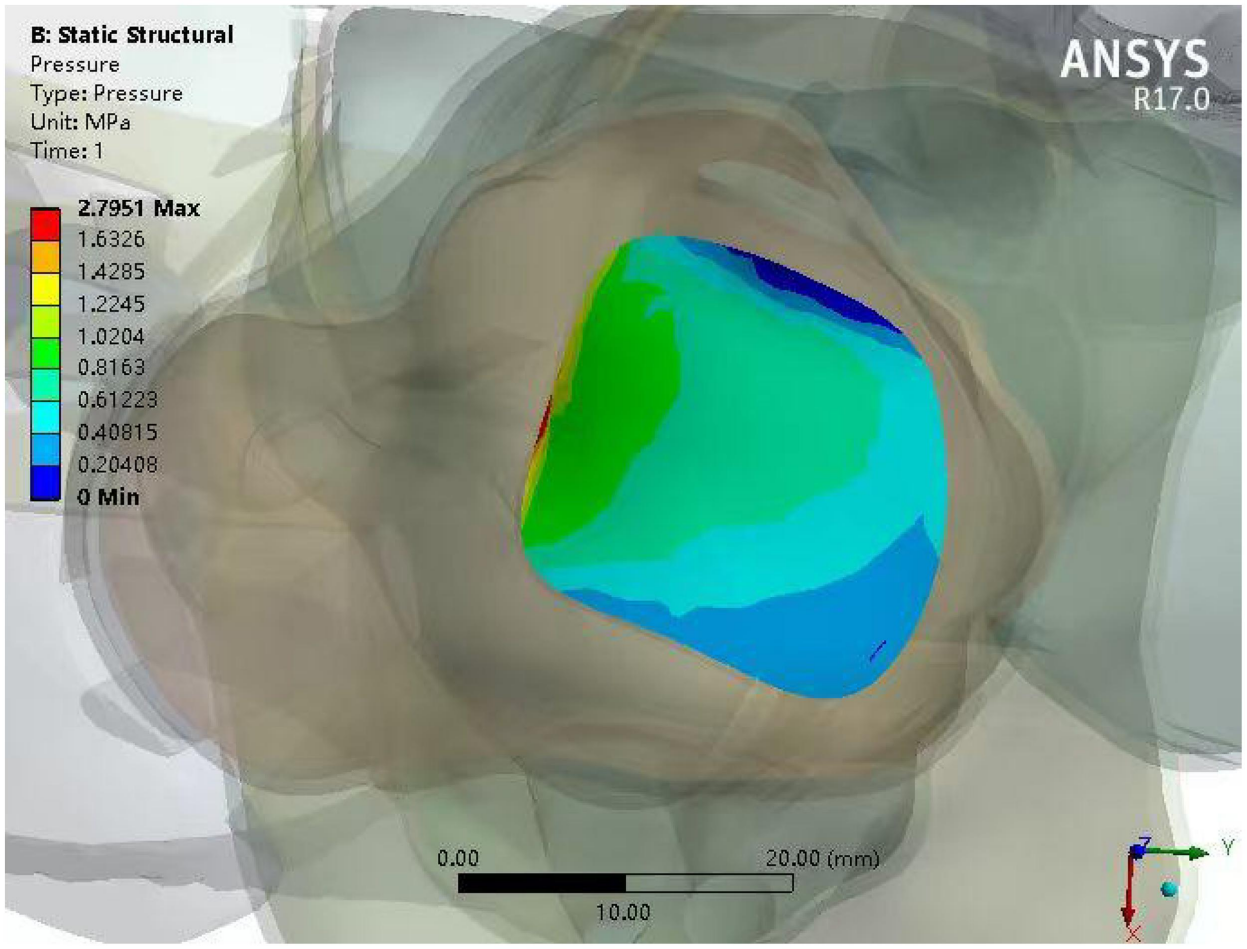
Contact stress cloud map of the tibial talar joint surface under 600 N load.

Under 600 N axial compression, the intact model converged without excessive element distortion or nonphysiologic contact penetration. The predicted peak tibiotalar contact stress was 2.80 MPa (Figure 4), which was close to the previously reported finite element value of 2.74 MPa and cadaveric benchmark value of 2.92 MPa (21). The spatial distribution of stress was concentrated over the anterolateral tibial plafond and corresponding lateral talar dome, with smooth peripheral decay and no obvious numerical artifacts. These findings supported the validity of the model for subsequent comparative analyses(Table 4).

**Table 4:** Validation of the intact ankle model under 600 N axial compression.

Reference dataset	Loading condition	Peak tibiotalar contact stress (MPa)	Difference from present model (MPa)	Relative difference (%)
Published cadaveric benchmark	600 N axial compression	2.9200	−0.1249	−4.3
Published finite element benchmark	600 N axial compression	2.7400	+0.0551	+2.0
Present finite element model	600 N axial compression	2.7951	–	–

### 3.2 Effect of residual syndesmotic instability

In the intact model, peak tibiotalar contact stress was 0.95 MPa, and distal fibular displacement measured 1.20 mm laterally and 3.69 mm posteriorly. After creation of residual syndesmotic instability by transection of the AITFL and IOL, peak contact stress increased to 1.30 MPa, representing a 36.8% increase relative to the intact model. Stress distribution shifted laterally, indicating loss of normal ankle mortise containment.

**Figure 5:**
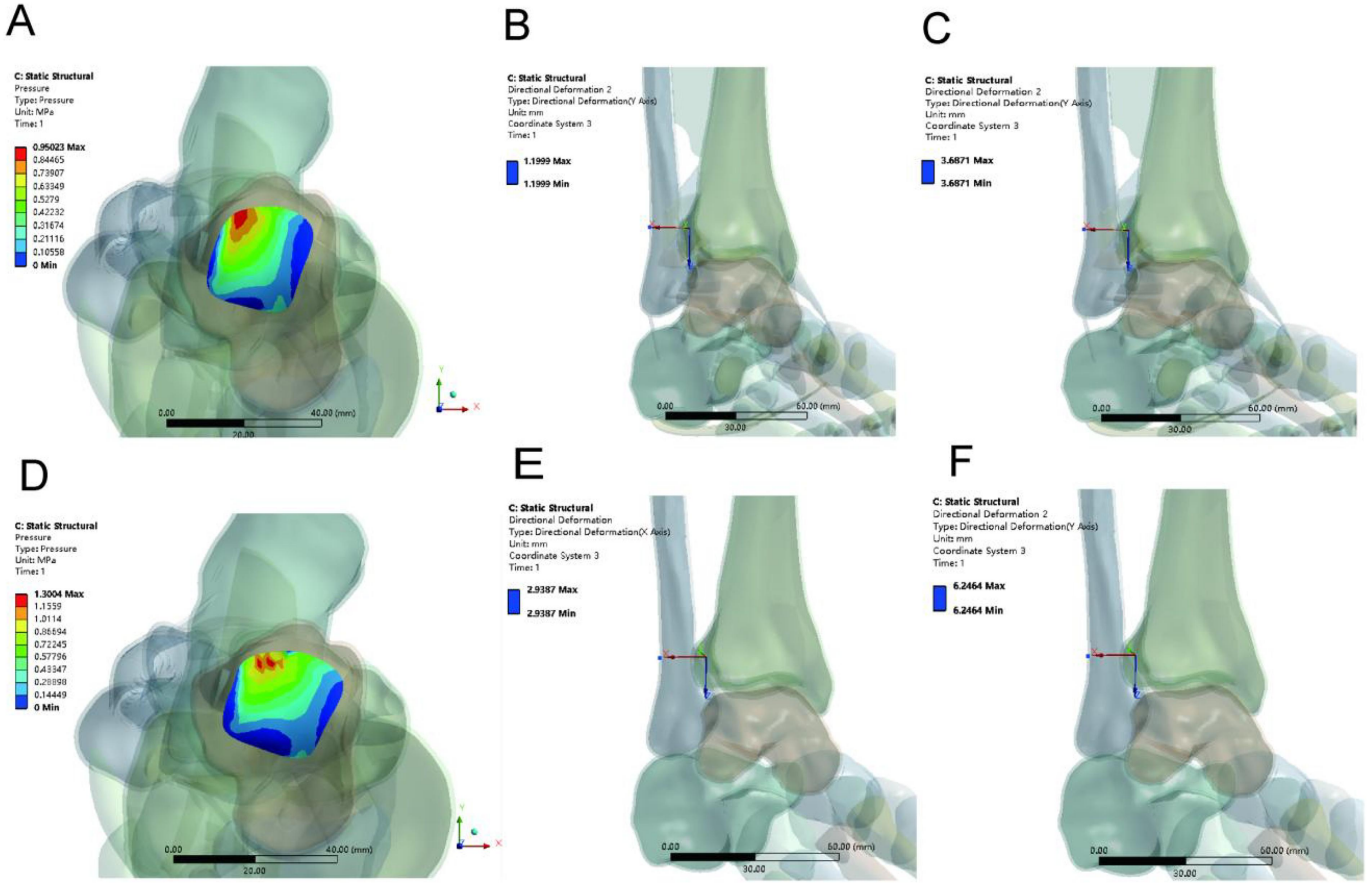
Effect of residual syndesmotic instability on tibiotalar contact mechanics and distal fibular displacement. **(A)** Contact-stress distribution in the intact ankle model. **(B,C)** Distal fibular displacement in the intact model. **(D)** Contact-stress distribution after creation of residual. **(E,F)** Distal fibular displacement in the instability model.

Residual instability also markedly increased fibular motion. Lateral displacement increased to 2.94 mm, and posterior displacement increased to 6.25 mm, far exceeding the physiologic range observed in the intact model. These findings indicate that residual syndesmotic instability substantially alters both joint contact mechanics and distal fibular kinematics (Figure 5 A–F).

### 3.3 Tricortical screw fixation

Across the nine tricortical screw configurations, peak tibiotalar contact stress ranged from 0.65 to 1.20 MPa, remaining well below the injured model and generally close to the intact state. However, fixation height had a strong effect on distal fibular control.

At 20 mm above the plafond, screw fixation restored biomechanics most closely to the intact model. Lateral displacement ranged from 0.98 to 1.13 mm, and posterior displacement ranged from 3.37 to 3.53 mm, indicating near-physiologic restoration of syndesmotic stability. At this height, changing the anterior tilt angle produced only small differences in fibular motion.

At 30 mm, screw fixation still provided acceptable control but allowed significantly more fibular motion than the 20-mm constructs (lateral displacement increased by 51%–68%, posterior displacement increased by 14%–33% compared to 20-mm screw fixation). Lateral displacement ranged from 1.65 to 1.73 mm, and posterior displacement ranged from 4.01 to 4.49 mm. Notably, peak tibiotalar contact stress remained low and comparable to the 20-mm group, indicating that moderate increases in fibular displacement at 30 mm did not translate to significant alterations in joint contact mechanics under the tested loading conditions.

At 40 mm, screw fixation became clearly less effective. Lateral displacement increased to 2.61–2.78 mm, and posterior displacement increased to 5.81–5.90 mm, approaching the values of the injured model. Although contact stress was still improved relative to the unstable state, the kinematic control of the distal fibula was substantially compromised.

**Figure 6:**
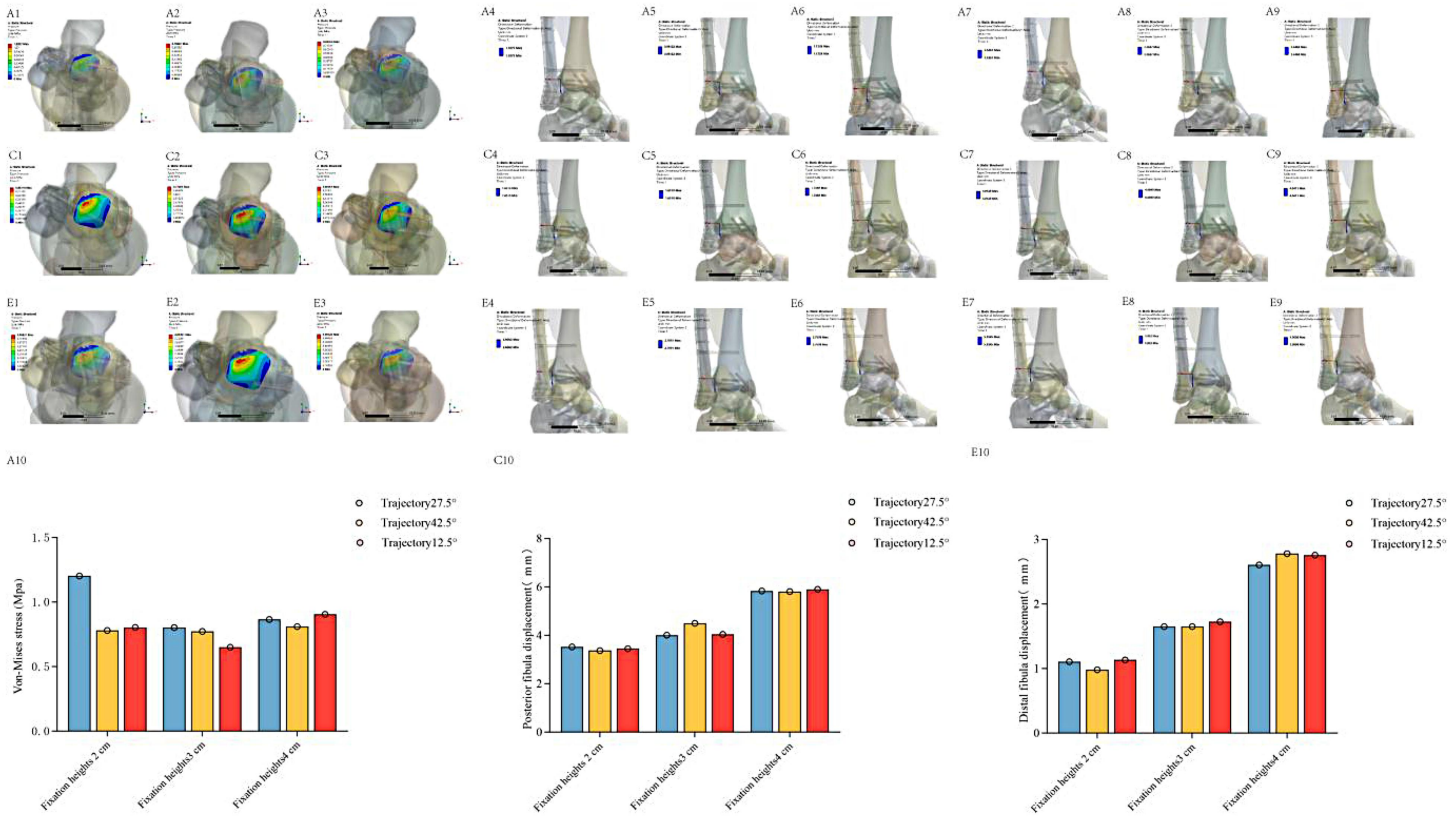
Biomechanical performance of single tricortical screw fixation across different fixation heights and anterior tilt angles.A1–A3, C1–C3, and E1–E3 show the maximum distal tibial articular contact stress for screw fixation at heights of 2, 3, and 4 cm, respectively, with anterior tilt angles of 27.5°, 42.5°, and 12.5°. A4–A6, C4–C6, and E4–E6 show the corresponding distal fibular lateral displacement, and A7–A9, C7–C9, and E7–E9 show the distal fibular posterior displacement. A10–C10 summarize the results as bar charts for tibiotalar contact stress, fibular lateral displacement, and fibular posterior displacement, respectively.

Peak implant von Mises stress in the screw models ranged from 93.5 to 244.3 MPa, with no configuration approaching the yield strength of titanium alloy under the simulated loading condition (Figure 6).

### 3.4 Suture-button fixation

Across the nine suture-button configurations, peak tibiotalar contact stress ranged from 0.80 to 0.95 MPa, closely approximating the intact ankle and showing less variation across groups than the screw models. However, fibular motion and implant stress were strongly influenced by fixation height and, in one case, by anterior tilt.

At 20 mm, all three suture-button constructs showed comparable biomechanical behavior. Lateral displacement ranged from 1.25 to 1.38 mm, and posterior displacement ranged from 4.23 to 4.49 mm, indicating greater micromotion than screw fixation but substantially more stability than the injured model. Within the 20-mm suture-button subgroup, differences among anterior tilt angles were modest: compared to the 27.5° configuration, the 12.5° configuration showed 7.0% less lateral displacement (1.25 vs. 1.35 mm) but 6.2% greater posterior displacement (4.49 vs. 4.23 mm), while the 42.5° configuration showed 2.2% greater lateral displacement (1.38 vs. 1.35 mm) and 4.5% greater posterior displacement (4.42 vs. 4.23 mm). All differences were less than 10% of the intact model values, indicating that anterior tilt angle had only a minor fine-tuning effect on fibular motion distribution rather than a fundamental impact on overall construct stability.

At 30 mm, fibular motion increased further. Lateral displacement ranged from 1.75 to 1.89 mm, and posterior displacement ranged from 4.73 to 5.03 mm, indicating moderate stability but more laxity than the 20-mm constructs.

At 40 mm, the suture-button constructs were biomechanically inadequate for syndesmotic control. Lateral displacement reached 2.90–2.91 mm, and posterior displacement reached 6.03–6.14 mm, nearly reproducing the injured state. Thus, although contact stress remained relatively favorable, the construct no longer effectively controlled syndesmotic separation at this height.

**Figure 7:**
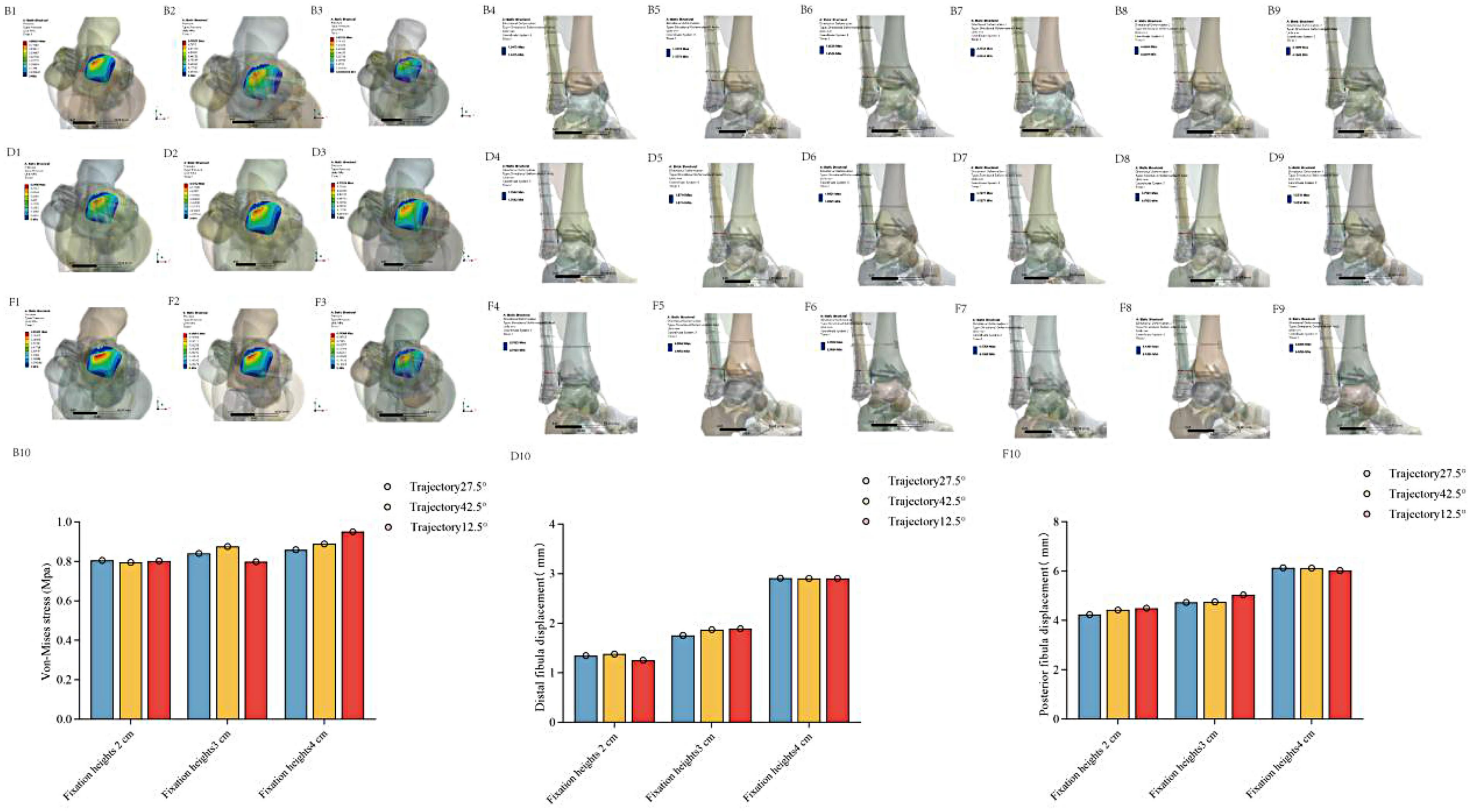
Biomechanical performance of suture-button fixation across different fixation heights and anterior tilt angles.B1–B3, D1–D3, and F1–F3 show the maximum distal tibial articular contact stress for suture-button fixation at heights of 2, 3, and 4 cm, respectively, with anterior tilt angles of 27.5°, 42.5°, and 12.5°. B4–B6, D4–D6, and F4–F6 show the corresponding distal fibular lateral displacement, and B7–B9, D7–D9, and F7–F9 show the distal fibular posterior displacement. B10–F10 summarize the results as bar charts for tibiotalar contact stress, fibular lateral displacement, and fibular posterior displacement, respectively.

Peak implant von Mises stress in the suture-button models ranged from 133 to 483 MPa, exceeding the levels observed in the screw models and showing the greatest concentrations in the 20-mm and 30-mm constructs (Figure 7).

### 3.5 Between-construct comparison

When the two fixation methods were compared directly, several patterns emerged. First, fixation height had a larger biomechanical effect than anterior tilt angle for both constructs. Second, both fixation methods were effective at 20–30 mm, whereas 40 mm fixation was consistently inferior. Third, at the same height, tricortical screws provided stronger restraint of distal fibular motion, while suture-button fixation preserved greater physiologic micromotion. Finally, the suture-button constructs generally experienced higher implant stress, indicating a different load-sharing pattern between the two fixation methods. It is important to note that these stress values represent instantaneous peak stresses under quasi-static loading, and no fatigue analysis or failure criteria were applied in the present study. Therefore, these results cannot be directly interpreted as predictions of clinical implant failure or longevity. Higher implant stress may indicate a greater susceptibility to fatigue failure under cyclic loading conditions, but this hypothesis requires validation through long-term cyclic testing and clinical outcome studies.

Among the tested configurations, the most balanced screw constructs were the 20-mm placements, whereas the 20-mm suture-button constructs also demonstrated similar and generally favorable biomechanical behavior. Within the 20-mm suture-button subgroup, differences among anterior tilt angles were modest, with the 12.5° configuration showing slightly less lateral displacement but slightly greater posterior displacement than the 27.5° and 42.5° configurations (Table 5).

**Table 5:** Peak tibiotalar contact stress and distal fibular displacement for all finite element model configurations.

Model code	Fixation type	Fixation height (mm)	anterior tilt angle (°)	Lateral displacement (mm)	Posterior displacement (mm)	Peak tibiotalar contact stress (MPa)
Intact	None	–	–	1.1999	3.6871	0.95023
Injured	None	–	–	2.9387	6.2464	1.3004
A3	Single tricortical screw	20	12.5	1.1326	3.4468	0.80454
A1	Single tricortical screw	20	27.5	1.1075	3.5261	1.2037
A2	Single tricortical screw	20	42.5	0.98122	3.3687	0.78021
C3	Single tricortical screw	30	12.5	1.7269	4.0411	0.65059
C1	Single tricortical screw	30	27.5	1.6515	4.0129	0.80318
C2	Single tricortical screw	30	42.5	1.6519	4.4949	0.77285
E3	Single tricortical screw	40	12.5	2.7598	5.9008	0.90526
E1	Single tricortical screw	40	27.5	2.6062	5.8345	0.86621
E2	Single tricortical screw	40	42.5	2.7815	5.8120	0.81091
B3	Suture-button	20	12.5	1.2536	4.4949	0.80312
B1	Suture-button	20	27.5	1.3475	4.2334	0.80503
B2	Suture-button	20	42.5	1.3773	4.4229	0.79571
D3	Suture-button	30	12.5	1.8921	5.0334	0.79834
D1	Suture-button	30	27.5	1.7542	4.7277	0.84060
D2	Suture-button	30	42.5	1.8714	4.7503	0.87520
F3	Suture-button	40	12.5	2.9034	6.0296	0.95066
F1	Suture-button	40	27.5	2.9123	6.1368	0.85921
F2	Suture-button	40	42.5	2.9052	6.1205	0.88857

## 4 Discussion

The most important finding of this study was that, under the tested loading conditions and within the parameter ranges evaluated, fixation height dominated the biomechanical response of syndesmotic fixation in a single-subject patient-specific model of Weber B ankle fracture with residual syndesmotic instability. Residual instability increased tibiotalar contact stress by nearly 37% and markedly increased distal fibular lateral and posterior translation, reinforcing the need for fixation when syndesmotic instability persists after fracture reduction. Both tricortical screw fixation and suture-button fixation restored near-normal joint mechanics when placed 20–30 mm above the tibial plafond, whereas fixation at 40 mm behaved much closer to the unstable state.

Notable differences exist in the selection of fixation techniques between the present study and prior biomechanical investigations. Specifically, the study referenced as PMID: 35732561 was designed as a head-to-head comparison of the overall efficacy between quadcortical screw fixation and the suture-button system (21). Internationally, the choice between tricortical and quadcortical screw fixation for syndesmotic injuries remains a subject of ongoing debate, with most published studies reporting comparable stabilizing efficacy between the two approaches. It is widely acknowledged that quadcortical fixation offers the advantage of easier retrieval of fractured screw fragments from the medial tibia in the event of implant breakage. However, tricortical screw fixation is more prevalently adopted in routine clinical practice in our setting. This technique avoids penetration of the medial tibial cortex, thereby reducing the risk of medial soft tissue irritation and periosteal injury, and does not require an additional medial surgical approach. Clinically, screws are routinely removed 8–12 weeks postoperatively before full weight-bearing to mitigate the risk of screw fracture. Accordingly, to address questions more relevant to local clinical practice and real-world surgical decision-making, the present study compared tricortical screw fixation with the suture-button system.

The pathologic changes observed in the injured model are consistent with classic ankle biomechanics. Even small disturbances in mortise congruity have been shown to alter contact mechanics substantially (8, 9). In the present study, transection of the AITFL and IOL caused both increased joint contact stress and major increases in fibular translation, supporting the concept that these structures are essential restraints against syndesmotic separation and rotational malalignment (5, 7). From a clinical perspective, these findings provide a biomechanical explanation for the poor outcomes associated with residual syndesmotic malreduction and the development of post-traumatic osteoarthritis (4, 20).

The second major finding was that 20-mm and 30-mm fixation planes were biomechanically effective, whereas 40-mm fixation was not. This supports current surgical practice favoring placement roughly 2–3 cm above the ankle joint line (17). The likely explanation is mechanical: as the fixation point is moved proximally, the unsupported distal fibula gains a longer lever arm relative to the talar dome and becomes more vulnerable to lateral and posterior displacement under rotational loading. Although some prior studies have discussed higher fixation positions (16, 22), the present model suggests that any benefit from reduced implant stress at high placement comes at the cost of inferior syndesmotic control, which is the more clinically important objective.

The comparison between constructs also revealed a clinically meaningful trade-off. Tricortical screw fixation better restricted fibular motion and maintained lower implant stress, indicating stronger immediate restraint. However, it tended to reduce physiologic syndesmotic motion more than the suture-button construct. Suture-button fixation, by contrast, restored contact stress while allowing greater micromotion, which may better reproduce normal syndesmotic behavior and may partly explain the favorable functional results reported in some clinical meta-analyses (14–16). That said, the higher implant stress observed in the suture-button models suggests that the price of dynamic fixation may be increased local device loading.

An additional finding was that, within the 20-mm suture-button subgroup, differences among anterior tilt angles were relatively small. The 20 mm/12.5° configuration did not demonstrate marked overconstraint; instead, it showed biomechanical behavior broadly comparable to the 27.5° and 42.5° configurations, with slightly lower lateral displacement and slightly greater posterior displacement. This pattern suggests that, under the present loading conditions and within the tested range of 12.5° to 42.5°, fixation height exerts a stronger influence on syndesmotic mechanics than anterior tilt angle. The biomechanical explanation for this observation is that fixation height determines the lever arm length of the distal fibula relative to the talar dome: as fixation height increases, the lever arm lengthens, leading to exponentially greater fibular displacement under rotational loading. In contrast, anterior tilt angle primarily affects the orientation of the implant relative to the syndesmotic ligaments and the direction of applied forces, which has a smaller impact on overall construct stability. It should be noted that the limited effect of anterior tilt angle observed in this study may be partially due to the relatively narrow range of clinically acceptable angles tested; extreme angles outside this range could potentially have a more significant impact on construct performance.

From a biomechanical perspective, the present findings suggest three mechanistic insights that may inform clinical decision-making. First, if residual syndesmotic instability persists after all malleolus fractures fixation, it should be stabilized. Second, the fixation plane should preferably be kept within 20–30 mm above the tibial plafond. Third, implant choice should be individualized: tricortical screws may be preferable when maximum restraint is needed, whereas suture-button fixation may be attractive when preserving controlled physiologic motion is a priority, provided tunnel anterior tilt is accurate. It is emphasized that these are biomechanical findings from a finite element model, not direct clinical evidence, and clinical decisions should always be individualized based on patient factors, injury pattern, and surgeon experience.

This study has several important limitations that must be considered when interpreting the results:

**1. Single-subject model limitation**: The study was based on a single patient-specific anatomy from a 27-year-old male volunteer. The results may not fully generalize to populations with different age, sex, bone mineral density, or ankle morphology. Future studies should include multiple subject models to assess the impact of anatomical variability on the findings.
**2. Material model simplification**: All biological tissues and implants were modeled as homogeneous, isotropic, linear elastic materials. This simplification does not capture the inherent anisotropy, nonlinearity, and viscoelasticity of ligaments, cartilage, and bone. In particular, ligaments and sutures exhibit tensile-only nonlinear behavior, which was not modeled here. These assumptions may overestimate the stiffness of soft tissues and alter predicted load-sharing patterns. A sensitivity analysis incorporating nonlinear material models is needed to quantify the impact of these simplifications.
**3. Injury model simplification**: Clinically, Weber type B ankle fractures with associated syndesmosis injuries often involve damage to the AITFL and IOL, whereas the PITFL is more robust and resistant to rupture. In cases with significant separation of the syndesmosis, posterior ankle fracture frequently replaces PITFL injury. Consequently, after reduction and fixation, only AITFL and IOL injuries remain. This injury model thus represents the predominant form of syndesmotic instability associated with most Weber type B ankle fractures.Nevertheless,the injury model was limited to transection of the AITFL and IOL, representing a specific pattern of syndesmotic instability. Clinical Weber type B fractures also involve more complex injury patterns, including injuries to the PITFL, deltoid ligament, and variable osseous instability, which were not included. The effect of these additional injuries on syndesmotic fixation mechanics requires further investigation.
**4. Loading condition limitation**: The study used a single quasi-static loading condition (200 N axial compression + 5 N·m external rotation) to simulate early postoperative partial weight-bearing. This does not reproduce the full cyclic complexity of daily activities or higher-demand loading conditions. The stability of the conclusions under different load intensities and loading modes should be evaluated in future studies.
**5. Interface assumption limitation**: All bone-implant interfaces were modeled as fully bonded, which reflects immediate postoperative mechanical stability but does not account for potential micromotion, loosening, or osseointegration that may occur over time. The long-term biomechanical behavior of the constructs under biological remodeling conditions remains unknown.
**6. Model validation limitation**: Validation was limited to peak tibiotalar contact stress in the intact ankle model. Additional validation of distal fibular displacement and implant stress against experimental cadaveric data was not performed, which reduces the certainty of these outcome measures. Nevertheless, the results regarding fibular displacement align with the trends reported in previous studies (23).
**7. Mesh convergence analysis**: A formal mesh convergence study was not conducted, although the mesh density and refinement strategy were consistent with previously validated ankle models. A comprehensive mesh convergence analysis will be included in future work to ensure numerical reliability.
**8. Interpretation limitation**: The results represent instantaneous biomechanical responses under controlled laboratory conditions and should be interpreted as mechanistic insights rather than direct clinical evidence. No fatigue analysis or failure criteria were applied, so implant stress results cannot be used to predict clinical failure rates.

## 5 Conclusion

Within the limitations of this single-subject finite element study under the tested loading conditions, the following conclusions can be drawn:

1. Residual syndesmotic instability (AITFL and IOL transection) in Weber B ankle fracture markedly increases tibiotalar contact stress (by 36.8%) and distal fibular displacement.
2. Both single tricortical screw fixation and suture-button fixation restore near-physiologic ankle biomechanics when placed 20–30 mm above the tibial plafond, whereas fixation at 40 mm provides inadequate control of fibular motion.
3. Fixation height has a greater biomechanical effect than sagittal anterior tilt angle within the tested range of 12.5° to 42.5°, with differences in anterior tilt angle producing only modest changes in the distribution of fibular motion.
4. There is a biomechanical trade-off between the two constructs: tricortical screws provide greater immediate restraint of distal fibular translation, whereas suture-button fixation preserves more physiologic micromotion at the cost of higher instantaneous implant von Mises stress.

These findings provide mechanistic insights that may inform surgical decision-making for syndesmotic fixation in Weber B ankle fractures, but clinical validation is required to confirm their applicability to real-world patient populations.

## Data Availability

The original contributions presented in the study are included in the article/Supplementary Material, further inquiries can be directed to the corresponding author.
